# Treatment of Incontinence of Urine

**Published:** 1867-09

**Authors:** Abbots Smith


### TREATMENT OF INCONTINENCE OF URINE.

By ABBOTS SMITH, M.D., M.R.C.P., &c.

Dr. Smith states in his interesting brochure *On Diabetes; and on Enuresis arising from Irritability, Weakness, or Inflammation of the Bladder and Urinary Organs*, that it should not be overlooked that a cure of enuresis will be greatly facilitated by certain moral and dietetic measures. For instance, any bad habit of not getting out of bed for the purpose of emptying the bladder at proper intervals should be counteracted. The quantity of fluids taken by the patient should be moderately restricted, particularly in the evening. This constitutes the real secret of the occasional success of the plan of treatment termed "Dietta Sicca," resorted to by some practitioners for the purpose of diminishing the excessive secretion. It consists in giving thick soups, bread, roast, or baked meat, fish without sauce, and dried fruits; the amount of liquid nourishment is gradually lessened, and the patient's thirst is assuaged, by the use of baths. This plan, however, is useless when enuresis depends on actual disease of the bladder or kidneys. The usual diet should be selected chiefly from articles of food which, although nutritious, are unstimulating to the kidneys or to the bladder, and which are not difficult to digest. Of these, none is so well adapted as milk. Amongst the most objectionable articles of diet may be enumerated all liquids which are taken when hot, especially tea, spices, pastry, salted and preserved meats, and most compound dishes. The general remedial measures of sea-bathing, change of air, and exercise, will prove the most useful in atonic, strumous cases; but the patient should be cautioned with respect to riding on horseback, which is productive of the disorder in many persons of a delicate organization, and will, when excessively indulged in, frequently render the affection serious, and almost intractable to medical treatment.—*Half-Yearly Abstract.*

